# A Review of the Anti-Inflammatory Effects of Rosmarinic Acid on Inflammatory Diseases

**DOI:** 10.3389/fphar.2020.00153

**Published:** 2020-02-28

**Authors:** Chunxu Luo, Lin Zou, Huijun Sun, Jinyong Peng, Cong Gao, Liuchi Bao, Renpeng Ji, Yue Jin, Shuangyong Sun

**Affiliations:** ^1^ College of Pharmacy, Dalian Medical University, Dalian, China; ^2^ Department of Internal Cardiovascular, The Second Affiliated Hospital of Dalian Medical University, Dalian, China; ^3^ Key Laboratory for Basic and Applied Research on Pharmacodynamic Substances of Traditional Chinese Medicine of Liaoning Province, Dalian Medical University, Dalian, China; ^4^ Research Center of Pharmacodynamic, Tianjin Institute of Pharmaceutical Research New Drug Evaluation Co., Ltd., Tianjin, China

**Keywords:** rosmarinic acid, inflammatory diseases, anti-inflammatory, mechanism, treatment

## Abstract

Inflammatory diseases are caused by abnormal immune responses and are characterized by an imbalance of inflammatory mediators and cells. In recent years, the anti-inflammatory activity of natural products has attracted wide attention. Rosmarinic acid (RosA) is a water-soluble phenolic compound that is an ester of caffeic acid and 3, 4-dihydroxyphenyl lactic acid. It is discovered in many plants, like those of the Boraginaceae and Lamiaceae families. RosA has a wide range of pharmacological effects, including anti-oxidative, anti-apoptotic, anti-tumorigenic, and anti-inflammatory effects. The anti-inflammatory effects of RosA have been revealed through *in vitro* and *in vivo* studies of various inflammatory diseases like arthritis, colitis, and atopic dermatitis. This article mainly describes the preclinical research of RosA on inflammatory diseases and depicts a small amount of clinical research data. The purpose of this review is to discuss the anti-inflammatory effects of RosA in inflammatory diseases and its underlying mechanism.

## Introduction

Inflammation is an integral part of innate immunity. It includes the body’s removal of harmful signals and the initiation of protective responses and tissue healing processes (Medzhitov, 2008). The inflammatory response is an essential physiological process that maintains the homeostasis of the immune system. Inflammation is divided into acute inflammation and chronic inflammation, both of them have a significant impact on human health (Yi et al., 2017). The acute inflammatory process is characterized by the rapid recruitment of granulocytes (i.e., neutrophils, eosinophils, and basophils) into the body (Maskrey et al., 2011). Disorders of sustained inflammatory stimuli or ablation stages can lead to chronic inflammation, including rheumatoid arthritis, systemic lupus erythematosus, silicosis, atherosclerosis, and inflammatory bowel disease (Sherwood and Toliver-Kinsky, 2004; Maskrey et al., 2011). Macrophages and T lymphocytes are the primary immune cells involved in chronic inflammation and generate cytokines and enzymes that result in tissue destruction, manifested as tissue fibrosis (Mohan and Gupta, 2018). Inadequate inflammation can cause continuous infection of pathogens, while excessive inflammation can lead to chronic or systemic inflammatory diseases (Guo et al., 2015).

The pattern-recognition receptors (PRRs) on immune cells sense “danger” from protein-associated molecular patterns (PAMPs) linked to a pathogen or from danger-associated molecular patterns (DAMPs) triggered by a large number of endogenous stress signals from the host (Rea et al., 2018). The interleukin 1 (IL-1) cytokine family (IL-1α, IL-1β, IL-18, IL-33, IL-36α, IL-36β, and IL-36γ) acts as DAMPs and stimulates sterile inflammation caused by necrosis and increases the inflammation with infection-related tissue damage (Martin, 2016). The cyclooxygenase (COX) and 5-lipoxygenase (5-LOX) pathways, which are metabolized by arachidonic acid (AA), produce highly pro-inflammatory lipid mediators that are involved in the classic signs of inflammation, including redness, fever, pain, swelling, and loss of function, which are designed to eliminate harmful and harmful stimuli (Rea et al., 2018). A family of important receptors that stimulate inflammation includes Toll-like receptors (TLRs). TLR4 signaling mediated by the linker MyD88 activates the transcription factor nuclear factor kappa B (NF-κB), thereby inducing gene expression of pro-inflammatory factors like tumor necrosis factor (TNF), IL-6, and IL-1β (Foley, 2015). These proteins play an important role in inflammatory diseases.

**Figure 1:**
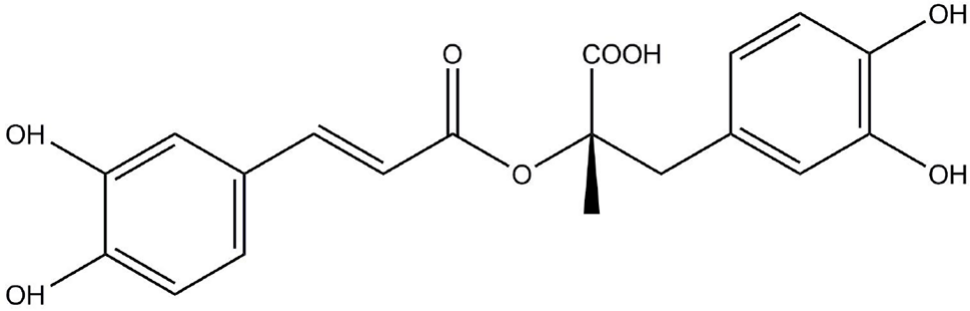
The structure of RosA.

Rosmarinic acid (RosA, **Figure 1**) is an ester of caffeic acid and 3, 4-dihydroxyphenyl lactic acid. It is usually discovered in species of the Boraginaceae family and the subfamily Nepetoideae of the Lamiaceae family (Petersen and Simmonds, 2003). RosA’s presence in medicinal plants, herbs, and spices is linked to beneficial and health-promoting effects (Ferreira et al., 2013). In plants, RosA is considered to be a cumulative defense compound, while in human, RosA has many biological activities, including antiviral, antibacterial, antioxidant, antimutagenic, and anti-inflammatory activities (Elufioye and Habtemariam, 2019). Many *in vitro* and *in vivo* studies have reported the anti-inflammatory effects of RosA in inflammatory diseases. This review systematically describes the therapeutic potential of RosA for inflammatory diseases and discusses its possible mechanisms.

## Effect of Rosmarinic Acid on Inflammatory Diseases

### Arthritis

Arthritis is an inflammatory disease that involves damage to one or more joints. It has more than one hundred types, the most common of which are osteoarthritis and rheumatoid arthritis (Petchi et al., 2013). Osteoarthritis (OA) is a progressive degenerative disease characterized by inflammation of the synovial, abrasion of the cartilage surface, subchondral sclerosis, and osteophyte generation, leading to loss of pain and movement (Yin et al., 2017). OA has long been considered as a degenerative disease of cartilage and is the only result of any process that causes increased pressure on a particular joint or the fragility of the cartilage matrix. The pathogenesis of OA is complicated and not fully understood, but an increasing number of researches have indicated that inflammation exerts a key role in the pathogenesis of OA (Berenbaum, 2013; Robinson et al., 2016). Rheumatoid arthritis (RA) is a chronic inflammatory autoimmune disease described as extensive infiltration and activation of inflammatory and mesenchymal cells, synovial cell proliferation, neovascularization, and occasional cartilage and bone destruction (Hur et al., 2007; Angelotti et al., 2017). RA is usually treated with nonsteroidal anti-inflammatory drugs (NSAIDs) and disease-resistant antirheumatic drugs (DMARDs), but they have adverse reactions, potential toxicity, and high cost, thus limiting their use. Presently, the field of arthritis study is rapidly developing in the direction of herbal research to find safe and effective drugs (Aloke et al., 2019).

OA is a multifactorial disease described primarily as the destruction of articular cartilage (Jiang and Tuan, 2015). Collagen 2 (COL2) and aggrecan (ACAN) are the main components of cartilage extracellular matrix (ECM) (Luo et al., 2017). The depletion of ACAN and COL2 results in the degradation of cartilage in OA (Mankin and Lippiello, 1970). A disintegrin and metalloproteinase with thrombospondin motifs (ADAMTS)-4 and ADAMTS-5 are responsible for ACAN depletion in osteoarthritic cartilage (Gendron et al., 2007). The inflammatory cytokine interleukin-1-beta (IL-1β) also exerts an important role in ECM degradation (Tu et al., 2017). An effect of RosA on OA has been reported in rat chondrocytes (Hu et al., 2018). In this experiment, chondrocytes were isolated from rat cartilage and incubated with RosA in the presence of IL-1β. RosA was found to inhibit IL-6 secretion and inhibit the gene and protein levels of ADAMTS-4 and ADAMTS-5. Moreover, RosA also inhibited the ACAN and COL2 gene expression induced by IL-1β. The results indicate that RosA can degrade ECM in OA and may have a therapeutic effect on OA. Another study found that drinking high-RosA spearmint tea can be a potential complementary treatment for OA pain relief (Connelly et al., 2014). The study indicated that taking high RosA tea for 16 weeks per day could significantly improve stiffness and physical disability scores in adults with knee OA and could significantly reduce pain.

T cells exert a crucial role in the development and progression of RA, and the apoptosis of potentially pathogenic T cells is considered to be an important therapeutic option. The study found that RosA can induce apoptosis in activated T cell subsets in RA patients by the mitochondrial pathway (Hur et al., 2007). The results showed that RosA induced CD3^+^CD25^+^ activated T cell apoptosis in 57.1% of RA patients in a dose-dependent manner, and RosA showed stronger apoptotic activity against the CD4^+^CD45RO^+^ effector T cell subset than the CD4^+^CD45RA^+^ naive T cell subset. In addition, RosA inhibited MMP destruction, reduced Bcl-2 expression, and induced Cyt c release from mitochondria to the cytoplasm. These results supported the view that RosA induced the apoptosis of activated T cells from RA patients through the mitochondrial pathway. Another experiment found that RosA can improve arthritis symptoms in the mouse model of collagen-induced arthritis (CIA) (Youn et al., 2003). RosA could significantly reduce the arthritis index and the number of affected paws. Histopathological images indicated that RosA inhibits synovitis, and synovial tissue of RosA-treated mice indicated a great reduce in the frequency of COX-2-expressing cells. Therefore, the administration of RosA in a clinical setting provided therapeutic effects in the treatment of RA. Furthermore, it has been reported that RosA extracted from pomegranate peel showed significant anti-arthritic potential in arthritis induced by Freund’s complete adjuvant (FCA) (Gautam et al., 2019).

### Colitis

Inflammatory bowel disease (IBD) is a chronic recurrent intestinal inflammation that includes Crohn’s disease (CD) and ulcerative colitis (UC). IBD is thought to be caused by an abnormal and sustained immune response to microorganisms in the intestine caused by the genetic susceptibility of the individual (Zhang and Li, 2014). UC is a chronic disease described as diffuse inflammation of the colon and rectal mucosa. The typical clinical symptom of UC is bloody diarrhea (da Silva et al., 2014). In contrast to CD, the inflammation of UC is limited to the colonic mucosa and the affected colon (Adams and Bornemann, 2013). In recent decades, the incidence of IBD has elevated in some areas of the world, especially in developing countries (da Silva et al., 2014). Consequently, the need for effective and safe natural compounds is increasing for IBD patients.

Studies have found that RosA can improve dextran sulfate sodium (DSS)-induced colitis (Jin et al., 2017; Zhao et al., 2018). In this study, RosA markedly decreased the disease activity index (DAI) and inhibited DSS-stimulated colon length shortening and splenomegaly in mice. RosA significantly improved inflammatory cell infiltration in the DSS-induced colitis model. RosA inhibited the induction of COX-2 and iNOS expression and the generation of IL-1β, IL-6, and IL-22. Immunohistochemistry analysis indicated that RosA significantly inhibited the expression of NF-κBp65 and pSTAT3 and their transport to the nucleus in the inflamed mucosa. Western blot analysis indicated that RosA inhibited the increase in NF-κB and STAT3-associated proteins in the colon of colitis mice. Another article showed the effect of RosA and black rice anthocyanin-rich extract (BRAE) alone or in combination on colitis induced by DSS (Zhao et al., 2018). At the appropriate dose, the combination of BRAE and RosA significantly reduced the DAI and inhibited the NO content, serum IL-1β and TNF-α expressions as well as IL-1β and TNF-α mRNA expression. Furthermore, the combination of BRAE and RosA exerted its anti-inflammatory activity through decreasing myeloperoxidase (MPO) and NO expressions as well as the mRNA levels of IL-6, IL-1β, and iNOS.

Some researchers have modified RosA to reduce its hydrophilicity, effectively inhibiting hypoxia-inducible factor-prolyl hydroxylase-2 (HPH) and causing the activation of hypoxia-inducible factor (HIF)-1 to exert anti-TNBS-induced colitis effects in rats (Jeong et al., 2015).

### Atopic Dermatitis

Atopic dermatitis (AD), also known as atopic eczema, is a chronic recurrent inflammatory skin disease (Avena-Woods, 2017). The clinical features of the disease are the exacerbation and relief of eczema skin, accompanied by inflammation, itching and flaking, desquamation, dry skin and susceptibility to skin bacteria, and mold infections (Cabanillas et al., 2017). The pathophysiology of AD is intricate and multifactorial, including barrier dysfunction, cell-mediated immune response changes, IgE-mediated hypersensitivity, and environmental factors (David Boothe et al., 2017). Currently, a lot of researchers are energetically trying to develop therapeutic drugs with great anti-inflammatory effects and few side reactions.

T cells play an important role in AD’s pathogenesis (Kurita et al., 2019). AD is a bipolar inflammatory skin disease that can be thought of as having two distinct stages. In the acute phase, AD skin lesions are infiltrated by CD4^+^ T cells, which chiefly secrete the Th2 cytokines IL-4, IL-5, and IL-13. However, in the chronic phase, Th1 cells secrete interferon-γ (IFN-γ) (Jin et al., 2009). Some researchers have reported that RosA can alleviate 2, 4-dinitrofluorobenzene (DNFB)-induced AD in NC/Nga mice and have revealed the mechanism of its involvement (Jang et al., 2011). The authors showed in this report that RosA could significantly inhibit the generation of IFN-γ and IL-4 by activated CD4^+^ T cells. RosA significantly inhibited the development of skin lesions and the thickness of the ear and reduced serum total IgE levels. RosA suppressed the infiltration of CD4^+^ T cells, CD8^+^ T cells, and mast cells into DNFB-induced skin lesions in NC/Nga mice. The above results indicated that RosA inhibited the development of AD-like dermatitis in DNFB-stimulated NC/Nga mice by decreasing the production of IFN-γ and IL-4 *via* activated T cells and the level of total serum IgE. Furthermore, RosA had the effect of improving AD in clinical studies (Lee et al., 2008). This effect was observed by the topical application of a RosA (0.3%) emulsion twice daily to the elbow flexion of 21 patients with mild AD. It was found that RosA could significantly reduce the statistically significant Severity Scoring of Atopic Dermatitis (SCORAD) score and reduce pruritus and transepidermal water loss (TEWL). In the patch test, no patient responded, indicating that RosA could be safely applied to human skin. These consequences indicate that RosA was able to be used as a therapeutic agent for AD. The author also suggested at the end of the article that RosA could improve AD symptoms by inhibiting IKK-β.

### Asthma

Asthma is a common chronic airway disease described as a complicated interaction between airway obstruction, bronchial hyperresponsiveness (BHR), and airway inflammation (Manuyakorn et al., 2013). Lots of cells and cellular components exert a role in this process, especially mast cells, eosinophils, T lymphocytes, macrophages, neutrophils, and epithelial cells (Mims, 2015).

RosA has been found to inhibit ovalbumin (Ova)-stimulated airway inflammation in a mouse model of asthma (Liang et al., 2016). The mitogen-activated protein kinases (MAPKs) regulate the synthesis and secretion of pro-inflammatory mediators during the inflammatory process, and their family contains three different stress-activated protein kinase pathways: p38, JNK, and ERK (Li et al., 2017). Constant activation of NF-κB has been found in allergic asthma, and the suppression of the NF-κB pathway attenuates asthma induced by OVA (Gu et al., 2017). In this experiment, RosA significantly inhibited the increase in inflammatory cells and Th2 cytokines in bronchoalveolar lavage fluid (BALF), decreased total IgE and Ova-specific IgE concentrations, and significantly improved airway hyperresponsiveness (AHR). Histological analysis showed that RosA significantly reduced the number of inflammatory cells and excessive mucus secretion in the airways. Pretreatment with RosA led to a significant decrease in AMCase, CCL11, CCR3, Ym2, and E-selectin mRNA levels in lung tissue and a significant regulation of NF-κB and MAPK activation. Therefore, this study suggested that RosA may be a promising candidate for asthma treatment. The protective effect of RosA may be through the inhibition of ERK, JNK, and p38 phosphorylation and the activation of NF-κB.

Another experiment evaluated the immunomodulatory effects of *Ocimum gratissimum* (Og) and RosA in a mouse respiratory allergy model caused by *Blomia tropicalis* (Bt) mite (Costa et al., 2012). This experiment found that RosA significantly inhibited the number of total inflammatory cells and eosinophils. Lung histopathology images showed that treatment with RosA reduced inflammatory cell infiltration around the bronchi and perivascular areas and inhibited mucus secretion in lung tissue. In addition, RosA could reduce the level of IL-4. The results of this experiment strongly supported the potential use of RosA as an anti-inflammatory drug for the treatment of allergic asthma. In addition, an article reported the effect of rosmarinus officinalis extracts on asthmatic subjected resistant to routine treatments (Mirsadraee et al., 2018). It was found that the Asthma Control Test (ACT) score displayed marked improvement after treatment with rosmarinus officinalis. Clinical evaluation revealed that cough, sputum generation, and wheezing were markedly improved in the rosmarinus officinalis group. At the same time, Exhaled Nitric Oxide (FENO) was significantly reduced after treatment with rosmarinus officinalis. The author finally concluded that rosmarinus officinalis and RosA had the potential to treat asthma.

### Allergic Rhinitis

Allergic rhinitis (AR) is a symptomatic nose inflammation caused by immunoglobulin E (IgE)-mediated endocardial inflammation (Bousquet et al., 2008). AR is one of the most common chronic diseases in the world, influencing 10 to 20% of the population (Devillier et al., 2014; Comert et al., 2016). Pollen is the biggest reported reason of seasonal allergic rhinoconjunctivitis (SAR) (Caillaud et al., 2015). Prospective researches indicate that SAR may be a predisposing factor for the development of asthma (Kopp et al., 2009).

RosA improved inflammation in the OVA-induced AR animal model (Oh et al., 2011). Administrate RosA could decrease elevated IgE levels in the serum, spleen, and nasal mucosa of OVA-sensitized mice. After treatment with RosA, the level of histamine in the serum was also significantly reduced. RosA suppressed the protein expressions and mRNA expressions of IL-1β, IL-6, and TNF-α in nasal mucosa or spleen as well. The article also found that the increase in mast cell and eosinophil infiltration caused by OVA sensitization was reduced in the drug-administered group. Furthermore, COX-2 expression and caspase-1 activity can be prevented by administering RosA in nasal mucosa tissue. In activated human mast cells, RosA suppressed the activation of NF-κB and caspase-1. The above consequences demonstrated the therapeutic potential of RosA for allergic rhinitis and allergic rhinoconjunctivitis.

Some researchers have studied whether oral RosA is effective in patients with SAR through clinical trials, and animal experiments have estimated the anti-inflammatory mechanism of RosA in the ear edema model (Osakabe et al., 2004). In clinical trials, the addition of RosA significantly reduced the rate of remission of each symptom compared with placebo. RosA markedly reduced the number of neutrophils and eosinophils in nasal lavage fluid as well. In the 12-tetradecanoylphorbol 13-acetate (TPA)-stimulated mouse ear edema model, the up-regulation of ICAM-1, VCAM-1 cyclooxygenase-2 (COX-2), keratinocyte chemoattractant (KC), and Macrophage inflammatory protein-2 (MIP-2) through TPA was significantly decreased by pretreatment with RosA. RosA can reduce neutrophil infiltration, as demonstrated by histological examination with hematoxylin-eosin staining. Another article also reported that RosA had a therapeutic effect on patients with SAR (Takano et al., 2004). Compared to placebo supplementation, supplementation with RosA-enriched *Perilla frutescens* extract led to a marked augment in responder rates for itchy nose, watery eyes, itchy eyes, and total symptoms. RosA could effectively treat mild SAR at least in part by inhibiting the infiltration of polymorphonuclear leukocytes (PMNLs) into the nostrils.

### Periodontal Diseases

Periodontal disease (PD) is thought to be a multifactorial disease caused by pathogenic infections, promoted by inflammation, and immune responses to bacteria, and altered by different environmental and genetic factors (Pietropaoli et al., 2010). The complex composition and organization of the periodontal ligament may be affected by damage to the steady-state equilibrium between the oral microbiome and the host, which may cause two main illnesses: gingivitis and periodontitis (Marty et al., 2017). Gingivitis is caused by microbial plaques that accumulate at or near the gingival sulcus. It was determined that gingivitis was mainly caused by B lymphocytes and plasma cells (Page, 1986). Gingivitis is thought to be an early periodontal disease that may or may not develop into periodontitis (Van Dyke et al., 1986). Periodontitis is a chronic inflammatory disease of dental support tissue with high incidence and alternating relief and acute exacerbation (Zhang et al., 2017). Periodontitis is described as persistent leukocyte infiltration, possibly regulated by the production of topical chemokine, but its pathogenesis has not been fully elucidated (Bostrom et al., 2015).

An article has reported the effects of the topical anti-inflammatory drugs ebselen and RosA on plaque-induced gingivitis progression in a rhesus monkey model (Van Dyke et al., 1986). Nonparametric indicators (G.I.) and plaque accumulation index (P.I.) were used to ascertain the extent of gingival inflammation and plaque accumulation. G.I. ranges from 0 (without erythema or edema) to 4 (serious erythema or edema, spontaneous bleeding, and ulceration). P.I. ranges from 0 (no plaque) to 4 (tooth completely covered by plaque). In this study, animals treated with RosA and ebselen showed lower G.I. and P.I. levels than controls. Therefore, the article drew a conclusion that, at least in the short term, RosA and ebselen were effective in decreasing gingival inflammation and plaque buildup when using a skin graft macaque model.

In addition, researchers have detected the effects of *Prunella vulgaris L.* (PVE) and its composition RosA on oxidative damage and inflammation in human gingival fibroblasts induced by LPS (Zdarilova et al., 2009). The pathogenesis of periodontitis is related to the imbalance of homeostasis between reactive oxygen species (ROS) and antioxidant defense systems (Liu C. et al., 2017). The authors pointed out that PVE and RosA reduced the production of ROS, the consumption of intracellular glutathione (GSH) and lipid peroxidation in LPS-treated cells. PVE and RosA not only inhibited the upregulation of IL-1β, IL-6, and TNF-α induced by LPS, but also inhibited the expression of iNOS. These results indicated that PVE and RosA may slow the progression of periodontitis by reducing the inflammatory response and the production of oxidative mediators in gingival fibroblasts.

### Acute Pancreatitis

Acute pancreatitis (AP) is an inflammatory disease described as acute inflammation and necrosis of the pancreatic parenchyma (Irrera et al., 2014). AP is considered to be a topical inflammatory response including premature intracellular activation of digestive enzymes in acinar cells, resulting in tissue self-digestion and possibly involving distant organs. Secretory acinar cells are considered to release chemokines and cytokines as well, which recruit white blood cells and trigger an inflammatory response that causes pancreatic edema and neutrophil accumulation (Makhija and Kingsnorth, 2002). The prognosis of AP patients depends largely on the incidence of organ failure and infected pancreatic necrosis. Despite the increasing incidence, there are currently no drugs to alleviate the symptoms of the disease and its course (Kambhampati et al., 2014).

RosA may have a protective effect on sodium taurocholate (NaTC)-induced AD (Fan et al., 2015). The pathology of NaTC-induced rat AP is very similar to that of severe acute pancreatitis (SAP) in humans, characterized by rapid onset of necrosis and inflammation of the pancreas and/or peripancreatic tissue. Inflammatory responses, pro-inflammatory cytokines like IL-1β, IL-6, and TNF-α, and the activation of NF-κB exert a crucial role in AP (Ma et al., 2018). The consequences indicated that RosA pretreatment markedly ameliorated pathological change in the pancreas; decreased serum amylase and lipase activity; decreased pancreatic myeloperoxidase activity; decreased systemic and pancreatic leukocyte IL-1β, IL-6, and TNF-α expression; and suppressed NF-κB translocation in the pancreas. RosA appeared to reduce the damage to AD caused by NaTC and reduced the release of inflammatory cytokines *via* suppressing the activation of NF-κB.

### Mastitis

Mastitis is a breast inflammation which is usually caused by a bacterial infection and is able to influence any mammal that is lactating (Sordillo, 2011). Human epidemiological studies have found that more than one-third of all lactating women develop mastitis, and the clinical manifestations of this illness are the main reason for mothers to stop breastfeeding. The development of mastitis is related to the extent to which the breast is exposed to bacterial pathogens. Multiple Gram-positive and Gram-negative pathogens lead to mastitis (Aitken et al., 2011). Gram-negative bacterial lipopolysaccharide (LPS) is considered to be an important factor in establishing animal models of inflammation (Gong et al., 2018).

Recently, an article reported the anti-inflammatory effect of RosA on LPS-induced mouse mastitis (Jiang et al., 2018). This experiment found that RosA treatment significantly improved mammary gland structural damage and decreased myeloperoxidase activity. ELISA and qPCR consequences showed that RosA reduced the level of TNF-α, IL-1β, and IL-6 in tissues and mMECs in a dose-dependent manner. TLRs are a vital class of pathogen recognition receptors. TLR4 is the most characteristic member of the TLR family and exerts a key role in the innate immune response to LPS infection (Mateu et al., 2015). A developing number of studies have shown that TLR4 can bring the production of inflammatory cytokines and regulate the activation of the NF-κB signaling pathway (Liao et al., 2018). RosA dose-dependently decreased TLR4 level in HEK293-mTLR4/mMD-2 cells, suggesting that RosA can interdict the inflammatory response by straight targeting TLR4. The levels of the downstream signaling factors MyD88, IRAK1, TRAF6, and IKKβ of the TLR4 pathway were also significantly reduced. Furthermore, the administration of RosA significantly suppressed the phosphorylation of IκB and the activation of p65. The DNA binding activity assay further confirmed the similar suppression of the nuclear translocation of NF-κB *via* RosA. Therefore, RosA can attenuate LPS-induced mastitis through suppressing the TLR4/MyD88/NF-κB signaling pathway.

### Effect of Rosmarinic Acid on Other Inflammatory Diseases

In addition, studies have found that RosA has been reported in Japanese encephalitis and neuritis. RosA reduced the mortality of murine infected with Japanese encephalitis virus (JEV). The viral load and pro-inflammatory cytokine levels of JEV-infected animals receiving RosA were significantly reduced 8 to 9 days after infection compared to the levels of untreated infected mice (Swarup et al., 2007). RosA greatly decreased the level of TLR4 and CD14 and the activation of NF-κB and NLRP3 inflammatory bodies, which is associated with anti-neuroinflammation (Wei et al., 2018).

## Anti-Inflammatory Effects of Rosmarinic Acid on Different Disease Models

In addition to its therapeutic effects in inflammatory diseases, RosA also exerts an anti-inflammatory effect on other diseases. The anti-inflammatory effects of RosA on different disease models are presented in **Table 1**.

**Table 1:** The anti-inflammatory effect of RosA on different disease models.

Experimental model	Major outcomes	References
**Extrahepatic cholestasis in rats**	BDL rats exhibit increased liver NF-κB/AP-1 activity, inflammatory cell infiltration/accumulation and cytokine formation, while RosA improved these symptoms of hepatitis. Dietary RosA supplementation was possibly beneficial in the matter of ameliorating cholestasis-related liver injury by mechanisms including the resolution of oxidative burden and down-regulation of HMGB1/TLR4, NF-κB, AP-1, and TGF-β1/Smad pathway.	(Lin et al., 2017)
**LPS-induced acute lung injury in mice**	RosA significantly reduced LPS-induced TNF-α, IL-6, and IL-1β production; RosA plays an anti-inflammatory effect on the acute lung injury in mice by inhibiting ERK/MAPK signaling in a dose-dependent manner.	(Chu et al., 2012)
**DEP-induced lung injury in mice**	RosA inhibited lung expression of KC, IL-1β, MCP-1 and MIP-1α, and inhibited the level of iNOS mRNA in the lung and the generation of nitrotyrosine and 8-OHdG. RosA inhibited DEP-stimulated lung injury through decreasing the level of pro-inflammatory molecules.	(Sanbongi et al., 2003)
**H_2_O_2_-induced NHDF damage**	RosA decreased NF-κB activity in NHDFs; RosA markedly dose-dependently reduced the levels of TNF-α and IL-6. RosA inhibited H_2_O_2_-induced inflammatory response in NHDFs.	(Hahn et al., 2017)
**Carrageenan-induced paw edema, liver I/R and thermal injury models in rat**	RosA could significantly reduce the increase in paw volume by inhibiting the inflammatory process associated with edema formation; RosA inhibited inflammatory processes associated with hepatic I/R, thereby reducing persistent liver damage following reperfusion; RosA reduced the systemic release of pro-inflammatory cytokines and reduced lung damage caused by scalding. The mechanism might be related to the activation of the NF-κB pathway and the suppression of MMP-9 activation.	(Rocha et al., 2015)
**Poly (I:C)-induced inflammatory reaction of epidermal keratinocytes**	RosA significantly inhibited the expression of IL-1β, IL-6, IL-8, CCL20, and TNF-α and down-regulated the NF-κB pathway. In the aspect of reducing the levels of NLRP3 and ASC and the secretion of activated IL-1β and caspase-1, RosA the inhibited poly (I: C)-induced activation of inflammatory bodies.	(Zhou et al., 2016)
**Type 1 diabetic mice**	RosA treatment reduced the levels of IL-6, TNF-α and PGE_2_ in the liver and the activity of COX-2. RosA might be an effective protective agent against liver damage in diabetes.	(Wen and Yin, 2017)
**CCl-induced neuropathic pain in rats**	RosA was able to prevent and attenuate CCl-stimulated behavioral characteristics in prophylactic and treatment groups, respectively. RosA inhibited the levels of TNF-α, iNOS, Iba-1, TLR-4, and GFAP. The anti-inflammatory effects of RosA might play an important role in the observed antinociceptive properties.	(Rahbardar et al., 2018)
**LPS-induced microglia activation in the N9 murine microglial cell line**	RosA attenuated the level of the M1-labeled iNOS and the expressions of pro-inflammatory factors, involving TNF-α, IL-1β, and IL-6. RosA inhibited the level of M2-labeled Arg-1 by inhibiting the activation of cleaved caspase-3. RosA attenuated microglial cells activation in N9 mice by downregulating the expression of inflammatory cytokines and caspase-3.	(Coelho et al., 2017)
**LPS-stimulated HUVECs**	The addition of RosA to HDF did not affect the morphology or viability of HUVECs and inhibited inflammatory responses stimulated by LPS, containing the level of IL-1β, IL-6, TNF-α, and iNOS, as well as NO generation. Using HDF to supplement RosA could reduce inflammation and ameliorate long-term treatment in patients with dialysis-induced renal failure.	(Wang et al., 2017)
**Cardiac I/R injury**	RosA pretreatment suppressed the expressions of inflammatory cytokines (IL-6, TNF-α, and CRP), upregulated PPARγ level and downregulated NF-κB level. RosA attenuated heart damage by activating PPARγ and downregulating NF-κB-mediated pathways, thereby suppressing inflammation and cardiomyocyte apoptosis in cardiac I/R injury models.	(Han et al., 2017)
**H22 tumor-bearing mice**	By adjusting the secretion of cytokines related to inflammation and angiogenesis and inhibiting the level of NF-κB p65 in the xenograft microenvironment, RosA could effectively inhibit tumor growth and had fewer toxic effects. RosA was a potential drug for the treatment of HCC.	(Cao et al., 2016)
**Unilaterally myringotomized Sprague-Dawley rats**	Topical and oral administration of RosA inhibited inflammation, reduced the thickness of the TM, and prevented spinal sclerosis in rats that had undergone tonsillectomies.	(Ozdemir et al., 2019)
**Aorta of diabetic rats**	RosA protected the aortic endothelium-dependent relaxation and ultrastructure and prevented damage caused by diabetes. The anti-inflammatory effects of RosA appeared to be involved in this protective mechanism.	(Sotnikova et al., 2013)
**MKN45 human gastric cancer cells and mouse xenograft models established using MKN45 cells**	RosA inhibited pro-inflammatory cytokines and microRNAs associated with inflammation, suggesting that RosA may inhibit Warburg effects through the inflammatory pathway, like IL-6/STAT3. MiR-155 was a key mediator of the relationship between inflammation and tumorigenesis. MiR-155 was a target gene that regulated the Warburg effect through inactivating the IL-6/STAT3 pathway. RosA inhibited the Warburg effect *in vivo*. RosA was a possible therapeutic drug that inhibited the Warburg effect of gastric cancer.	(Han et al., 2015)
**Ovariectomized rats treated with D-galactose**	RosA exerted an anti-inflammatory effect and inhibited the synthesis of PGE_2_, thereby benefiting the treatment of AD. RosA treatment reduced memory impairment by improving oxidative stress and inflammatory responses and was a potential candidate for slowing the progression of the disease.	(Kantar Gok et al., 2015)
**CP-induced nephrotoxicity**	RosA inhibited the expression of NF-κB and TNF-α, indicating that inflammation was inhibited. RosA improved renal oxidative stress, inflammation and apoptosis induced by CP. RosA’s renal protective activity might be at least partially due to a decrease in CYP2E1 level.	(Domitrovic et al., 2014)
**Carrageenan-induced paw edema model of inflammation in rats**	BME (rich in RosA) significantly impeded the onset of inflammation and dose-dependently reduced paw edema at 4 hours. BME treatment markedly decreased the generation of IL-1β and TNF-α. The article suggested that RosA and similar phenolic compounds could be used to treat inflammation-related damage.	(Usha et al., 2014)
**TSLP-stimulated human mast cell line, HMC-1 cells, and short ragweed pollen-induced allergic conjunctivitis mouse model**	RosA inhibited TSLP-induced mast cell proliferation *via* down-regulating MDM2 and up-regulating p53. RosA significantly reduced the generation of TNF-α, IL-1β and IL-6 stimulated by TSLP in HMC-1 cells. It also prevented the generation of pro-inflammatory cytokines in the EAC *in vivo* model and decreased the expressions of IgE, TSLP, and IL-4. The article showed that RosA had a great anti-inflammatory effect on TSLP-stimulated inflammatory responses. RosA could be used to treat allergic inflammation caused by a rise in the number of mast cells.	(Yoou et al., 2016)
**LPS-stimulated BMDCs**	RosA reduced the migration of cells by inducing expression of LPS-induced mature BMDC-specific chemokine receptors. RosA markedly decreased the expression of MCP-1 and MIP-lα in LPS-stimulated BMDCs and inhibited the activation of MAPK and the nuclear translocation of NF-κB stimulated by LPS. RosA had attractive new pharmacological properties that inhibited the LPS-induced upregulation of inflammatory chemokines in BMDCs.	(Kim et al., 2008)
**UVB-induced HaCaT cells**	When used simultaneously with radiation, RosA significantly reduced the expression of UVB-induced IL-6, IL-8, MCP-1, and TNF-α. RosA could avoid and/or limit the inflammatory cascade induced by UVB by reducing pro-inflammatory mediators and enhancing IL-10 and its protective function.	(Lembo et al., 2014)
**LPS- and CLP‐mediated HMGB1 release**	RosA effectively inhibited the release of HMGB1 in human endothelial cells and downregulated HMGB1-dependent inflammatory responses; RosA supressed HMGB1-mediated high permeability and leukocyte migration in mice; RosA decreased CLP-stimulated HMGB1 release and sepsis-related mortality. The article suggested that RosA ought to be considered as a candidate therapeutic for treating all kinds of inflammatory diseases by inhibiting the HMGB1 pathway. RosA was a potential treatment for curing serious vascular inflammatory diseases like sepsis and septic shock.	(Yang et al., 2013)
**MPTP-induced Parkinson’s disease in mice and MPP^+^ and α-synuclein-induced Parkinson’s disease in BV-2 cells**	RosA treatment could improve the motor function of Parkinson’s disease mice, increase the number of tyrosine-hydroxylase-positive cells, reduce the generation of pro-inflammatory cytokines, and inhibit the activation of microglia in the ventral midbrain; RosA reduced MPP^+^- or α-synuclein-induced secretion of pro-inflammatory cytokines; RosA administration decreased the levels of HMGB1, TLR4, and Myd88 in Parkinson’s disease animals and cell models and inhibited NF-κB nuclear expression. RosA could attenuate the inflammatory response by inhibiting the HMGB1/TLR4/NF-κB pathway, which might be beneficial for its activity against Parkinson’s disease.	(Lv et al., 2019)
**CLP-induced sepsis in rats and LPS-induced RAW264.7 cells**	RosA down-regulated the expressions of TNF-α, IL-6, and high-mobility box-1 proteins *in vivo*, suppressed the IκB kinase pathway, and regulated NF-κB. Intravenous injection of RosA alone or in combination with imipenem could downregulate serum TNF-α, IL-6, high-mobility group box 1 protein, triggering receptor expressed on myeloid cells and endotoxin and upregulate IL-10 serum levels. RosA had the ability to inactivate the inflammatory response in sepsis. RosA’s anti-inflammatory mechanism might be related to inhibition of the IκB kinase activity and thus inhibition of NF-κB signaling pathway activation.	(Jiang et al., 2009)
**Acute lung injury in mice infected with influenza virus**	RAG could regulate the expression of inflammatory cytokines induced by influenza virus, especially by reducing the expression of Th1 cytokines IFN-γ and TNF-α and increasing the expression of Th2 cytokines IL-4 and IL-5. After RAG administration, cell migration and infiltration were similarly reduced. RAG had a pleiotropic effect on viral pneumonia.	(Liu J.X. et al., 2017)
**DOX-induced neurotoxicity in rats**	RosA treatment ameliorated pro-inflammatory cytokines like TNF-α and iNOS and reduced oxidative stress biomarkers and brain monoamines. RosA could effectively prevent DOX-induced neurotoxicity, and the underlying mechanism of neuroprotection was possibly related to its antioxidant, anti-inflammatory, and anti-apoptotic effects.	(Rizk et al., 2017)
**Spinal cord injury**	RosA prevented the rise in the SCI-induced nuclear localization of NF-κB and the corresponding reduction in the nuclear localization of Nrf-2. RosA inhibited neuronal apoptosis through targeting ROS and inflammatory responses in SCI.	(Shang et al., 2017)
**Experimental diabetes with cerebral ischemia**	RosA inhibited NF-κB activation and decreased HMGB1 expression *in vitro* and *in vivo*. RosA protected the brain against I/R injury by attenuating diabetic brain I/R injury and alleviating the destruction of the BBB, and its protective effect might include the HMGB1 and NF-κB pathways. RosA had therapeutic potential as an anti-inflammatory lead compound useful in primary brain I/R injury in diabetes.	(Luan et al., 2013)
**Oxaliplatin-induced peripheral neuropathy in rats**	AMPK activation could be involved in oxaliplatin-induced mitochondrial dysfunction and glial-cell-mediated inflammation, thereby reducing OIPN. RosA relieved neuropathic pain caused by oxaliplatin through preventing mitochondrial dysfunction and glial-cell-mediated inflammation.	(Areti et al., 2018)
**Carrageenan-induced paw edema and cotton-pellet-induced granuloma formation in two mouse models**	RosA significantly inhibited carrageenan-stimulated paw edema and inhibited cotton-pellet-induced granuloma formation. RosA had central and peripheral antinociceptive activity and had an anti-inflammatory effect on acute and chronic inflammation. The article highlighted the potential use of RosA in relieving pain and treating inflammatory diseases.	(Boonyarikpunchai et al., 2014)
**Cadmium-induced ototoxicity**	RosA suppressed Cd^+^-induced cell death, ROS production, IL-6 and IL-1β increase, cyt c release, caspase-3 activation, and AIF translocation into the nucleus of auditory cells. RosA offset ototoxicity *via* inhibiting the damage of the hair cell arrays in the rat organ of Corti primary explants. The protective effect of RosA occurs by modulating inflammatory cytokines in cadmium-induced ototoxicity.	(Kim et al., 2013)

## Conclusion

Inflammation is an acute reaction to infection and tissue lesion to prevent damage to the body (Strowig et al., 2012). Excessive inflammation can lead to chronic or systemic inflammatory diseases. In the past few decades, the prevalence of inflammatory diseases has been on the rise, especially in developed countries (Xin et al., 2019). Rosmarinic acid is a class of aqueous phenolic compounds. Many reports have demonstrated that RosA has an important role in treating inflammatory diseases through multiple mechanisms, and RosA exerts anti-inflammatory effects to treat various diseases. Of all retrieved articles, we found that there was less clinical data of RosA in inflammatory diseases. Therefore, we mainly outlined the therapeutic potential of RosA in inflammatory diseases and its possible mechanisms in preclinical research. We hope that this review can obtain a reference for the future treatment of inflammatory disorders.

## Author Contributions

All the authors have written, reviewed the manuscript and agreed the ultimate version.

## Funding

This work was supported by Liaoning provincial department of education project (LZ2019013) and Special Program of Talents Development for Excellent Youth Scholars in Tianjin (TJTZJH-QNBJRC-2-7).

## Conflict of Interest

SS is employed by Tianjin Institute of Pharmaceutical Research New Drug Evaluation Co Ltd.

The remaining authors declare that the research was conducted in the absence of any commercial or financial relationships that could be construed as a potential conflict of interest.

## Abbreviations

BDL, Bile-duct ligation; BBB, Blood-brain barrier; BMDCs, Bone-marrow-derived dendritic cells; CLP, Cecal ligation and puncture; CCI, Chronic constriction injury; CP, Cisplatin; CRP, C-reactive protein; DEP, Diesel exhaust particles; DEP, Doxorubicinx; EAC, Experimental allergic conjunctivitis; GFAP, Glial fibrillary acidic protein; HDF, Hemodialysis fluid; HCC, Hepatocellular carcinoma; HMGB1, High-mobility group box 1; Iba-1, Ionized calcium-binding adapter molecule 1; I/R, Ischemia-reperfusion; BME, Methanolic leaf extract of *Baccaurea ramiflora*; MCP-1, Monocyte chemoattractant protein-1; NHDFs, Normal human dermal fibroblasts; OIPN, Oxaliplatin-induced peripheral neuropathy; PGE_2_, Prostaglandin E_2_; RAG, Rosmarinic acid-4-O-β-D-glucoside; STAT3, Signal transducer and activator of transcription-3; SCI, Spinal cord injury; OGD, Oxygen-glucose deprivation; TSLP, Thymic stromal lymphopoietin; TM, Tympanic membrane; MPP^+^, 1-methyl-4-phenylpyridinium; MPTP, 1-Methyl-4-phenyl-1, 2, 3, 6-tetrahydropyridine; 8-OHdG, 8-Hydroxyguanosine.
